# Pro-oncogene *Pokemon *promotes breast cancer progression by upregulating *survivin *expression

**DOI:** 10.1186/bcr2843

**Published:** 2011-03-10

**Authors:** Xuyu Zu, Jun Ma, Hongxia Liu, Feng Liu, Chunyan Tan, Lingling Yu, Jue Wang, Zhenhua Xie, Deliang Cao, Yuyang Jiang

**Affiliations:** 1 Guangdong Provincial Key Laboratory of Chemical Biology, Graduate School at Shenzhen, Tsinghua University, Lishui Road, Shenzhen 518055, People's Republic of China; 2 Institution of Clinical Medicine, First Affiliated Hospital of University of South China, Chuanshan Road, Hengyang 421001, People's Republic of China; 3 Department of Medical Microbiology, Immunology and Cell Biology, Simmons Cancer Institute, Southern Illinois University School of Medicine, 913 North Rutledge Street, Springfield, IL 62794, USA; 4 School of Medicine, Tsinghua University, Beijing 100084, People's Republic of China

## Abstract

**Introduction:**

Pokemon is an oncogenic transcription factor involved in cell growth, differentiation and oncogenesis, but little is known about its role in human breast cancer. In this study, we aimed to reveal the role of Pokemon in breast cancer progression and patient survival and to understand its underlying mechanisms.

**Methods:**

Tissue microarray analysis of breast cancer tissues from patients with complete clinicopathological data and more than 20 years of follow-up were used to evaluate *Pokemon *expression and its correlation with the progression and prognosis of the disease. DNA microarray analysis of MCF-7 cells that overexpress *Pokemon *was used to identify *Pokemon *target genes. Chromatin immunoprecipitation (ChIP) and site-directed mutagenesis were utilized to determine how Pokemon regulates *survivin *expression, a target gene.

**Results:**

Pokemon was found to be overexpressed in 158 (86.8%) of 182 breast cancer tissues, and its expression was correlated with tumor size (*P *= 0.0148) and lymph node metastasis (*P *= 0.0014). *Pokemon *expression led to worse overall (*n *= 175, *P *= 0.01) and disease-related (*n *= 79, *P *= 0.0134) patient survival. DNA microarray analyses revealed that in MCF-7 breast cancer cells, Pokemon regulates the expression of at least 121 genes involved in several signaling and metabolic pathways, including anti-apoptotic survivin. In clinical specimens, *Pokemon *and *survivin *expression were highly correlated (*n *= 49, *r *= 0.6799, *P *< 0.0001). ChIP and site-directed mutagenesis indicated that Pokemon induces *survivin *expression by binding to the GT boxes in its promoter.

**Conclusions:**

Pokemon promotes breast cancer progression by upregulating *survivin *expression and thus may be a potential target for the treatment of this malignancy.

## Introduction

Pokemon, also referred to as a factor that binds to inducer of shot transcript 1 (FBI) or leukemia/lymphoma-related factor (LRF), is the product of the *ZBTB7 *gene [1]. Pokemon is a pro-oncogenic protein overexpressed in lung cancer, diffuse large B-cell lymphomas (DLBCLs), non-Hodgkin's lymphoma (NHL), liver cancer, follicular lymphomas and breast cancer [1-4]. In animals, Pokemon was found to induce cell transformation by repressing the tumor suppressor ARF/p53 pathway [1]. In addition, Pokemon is implicated in transactivation of the human immunodeficiency virus (HIV)-1 *Tat *gene, adipogenesis, osteoclastogenesis and fatty acid synthesis [5-8]. However, the expression and role of *Pokemon *in human breast cancer remains unclear.

Pokemon functions as a transcription regulator with active roles in cell growth, differentiation and oncogenesis [2,9,10]. Pokemon interferes with GC box recognition by Sp1 via interacting with the zinc finger DNA binding domain, resulting in the repression of ADH5/FDH transcription [11]. Pokemon also affects the transcription of nuclear factor (NF)-κB-responsive genes by associating with the p65 subunit and inducing its nuclear import and stabilization [12]. The target genes of Pokemon include extracellular matrix collagen types I, II, IX, X and XI; aggrecan; fibronectin; elastin; cartilage oligomeric matrix protein (*COMP*); alcohol dehydrogenase *ADH5*/*FDH*, *ARF *and *Rb *tumor suppressors; and c-fos and c-myc oncoproteins [9,11-15]. In this study, we found that in breast cancer cells, Pokemon stimulates *survivin *expression by binding to its promoter.

Survivin, a member of the inhibitor of apoptosis proteins (IAP) [16], plays an important role in cell apoptosis and mitotic regulation. *Survivin *is expressed in fetal and cancer cells, but not in normal adult cells. *Survivin *is highly expressed in breast, colorectal, lung, gastric and bladder cancers, as well as in melanoma, hepatocellular carcinoma and malignant lymphoma, and its expression in these cancers is associated with poor clinical prognosis [17-25]. Much effort has been made to understand the regulatory mechanism of *survivin *expression. Various studies have shown that *survivin *expression is regulated by multiple oncogenes, tumor suppressors and growth factors, such as p53, Sp1, Krüppel-like factor 5 (KLF5) and epidermal growth factor receptor (EGFR) [26-30]. In the present study, we found that *survivin *expression is correlated with *Pokemon *expression in human breast cancer cells and demonstrated that Pokemon induces its expression by binding to the GC boxes in its promoter.

## Materials and methods

### Tissue microarray and clinical data

Microarrays of human breast carcinomas were provided by the Yale Cancer Center Critical Technologies group. Two microarrays were used in this study: Yale Tissue Microarray (YTMA) -23, containing 246 breast cancer cases with complete clinical records and more than 30 years of follow-up (Additional file 1), and YTMA-89, consisting of 54 recurrent breast cancer cases. Paraffin-embedded, formalin-fixed specimens of breast carcinoma tissue were identified from the archives of the Yale University Department of Pathology as available from 1961 to 1983. Complete treatment information was unavailable for the entire cohort of 246 primary breast cancer cases, but most patients were treated with postsurgical local radiation. None of the node-negative patients were given adjuvant systemic therapy. Among the node-positive patients, approximately 15% were given chemotherapy primarily consisting of adriamycin, cytoxan and 5-fluorouracil, and some were given tamoxifen (post-1978) [31,32]. Another two adjacent arrays composed of 50 breast cancers with matching normal adjacent tissues were obtained from Cybrdi, Inc. (CC08-1-07; Cybrdi, Inc., Rockville, MD, USA). Ethical approval for this study was obtained from the human research ethical advisory committee of Tsinghua University.

### Cell culture

The human breast cancer cells MCF-7 and MDA-MB-231 (American Type Culture Collection, Manassas, VA, USA) were maintained in Dulbecco's modified Eagle's medium (DMEM) supplemented with 10% heated fetal bovine serum (FBS), 2 mM glutamine, 100 U/ml penicillin and 100 μg/ml streptomycin at 37°C and 5% CO_2_.

### Immunohistochemistry

After dewaxing and hydration, tissue microarray slides were immersed into preheated citric acid buffer (pH 6.5) at 90°C to 95°C for 20 minutes with microwaving. After being blocked with 5% horse serum for 30 minutes, slides were incubated with anti-Pokemon or anti-survivin antibodies (1:50; Abcam, Cambridge, MA, USA) at 4°C in a humid box overnight. Thereafter slides were washed three times and then incubated with horseradish peroxidase-conjugated secondary antibody (1:800; Pierce Biotechnology, Rockford, IL, USA) at room temperature for 1 hour. Enhanced 3,3'-diaminobenzidine staining buffer (Pierce Biotechnology) was used to visualize signals. Staining intensity was evaluated blindly by at least one researcher and one pathologist and was scored from 0 to 3, representing negative and low, intermediate and high staining, respectively.

### Plasmid construction

*Pokemon*-expressing plasmid was generated by inserting the encoding region into pcDNA3.1 expression vector (Invitrogen, Carlsbad, CA, USA) at the site of the *Hin*dIII restriction enzyme. *Pokemon *primer pairs were 5'-CTT AAG CTT GCC ACC ATG GCC GGC GGC GTG G-3' and 5'-GTC AAG CTT TTA GGC GAG TCC GGC TGT GAA GTT AC-3'. *Survivin *promoter was subcloned into pGL4.10 basic plasmid (Promega, Madison, WI, USA) at the *Bam*HI and *Eco*RV restriction enzyme sites to drive luciferase expression. The amplification primer pairs were 5'-GTC AGA TCT AGT GAA AAG GAG TTG TTC CTT TCC TCC CTC-3' and 5'-GTC AAG CTT GCC GCC GCC GCC ACC TC-3' for a 2,082-bp fragment, 5'-GTC AAG CTT GCC GCC GCC GCC GCC ACC TC-3' and 5'-GTC AGA TCT AAA GAC AGT GGA GGC ACC AGG C-3' for a 1,054-bp fragment, and 5'-GTC AAG CTT GCC GCC GCC GCC ACC TC-3' and 5'-GTC AGA TCT TTG GGA TTA CAG GCA TGC ACC AC-3' for a 441-bp fragment.

### Site-directed mutagenesis

Mutants (pLuc-95 m and pLuc-231 m) of *survivin *promoter were generated using an *in vitro *site-directed mutagenesis system (Promega). The GGGTG sequence in the binding site of Pokemon was replaced by AAAAA and confirmed by sequencing.

### cDNA microarray analysis

Human cDNA microarrays covering 22 kb cDNA spots (CapitalBio, Beijing, China) were used. In brief, total RNA was extracted from MCF-7 cells with ectopic *Pokemon *expression and vector control using the TRIzol reagent (Invitrogen), and fluorescence-labeled cDNA probes were made for hybridization using 30 μg of total RNA with an oligo(dT)18 primer and SuperScript II Reverse Transcriptase (Gibco BRL, Carlsbad, CA, USA). Hybridized slides were scanned using a LuxScan 10K-A confocal laser microscopy scanner, and signal intensities for each spot were calculated by subtracting local background using LuxScan 3.0 software (CapitalBio). Three independent replicates were conducted, and the spot with ≥2.0-fold increase or decrease was considered a significant change. Gene expression signaling pathways were analyzed with MAS2.0 software (CapitalBio) [GEO:GSE27442].

### *Pokemon *silencing and Western blot analysis

*Pokemon *expression in MDA-MD-231 breast cancer cells was knocked down using chemically synthesized small interfering RNA (siRNA) siRNA#1 (targeting 426 to 444 bp, sense 5'-GCU GGA CCU UGU AGA UCA Att-3', and antisense 5'-UUG AUC UAC AAG GUC CAG Ctt-3'), siRNA#2 (targeting 476 to 494 bp, sense 5'-AGU ACC UCG AGU UCU UCC Att-3', and antisense 5'-UGG AAG AAC UCG AGG UAC Utt-3') [13] and siRNA#3 (targeting 624 to 642 bp, sense 5'-GGA GUA CCU CGA GUU CUU Ctt-3', and antisense 5'-GAA GAA CUC GAG GUA CUC Ctt-3'). The siRNA were introduced as described previously [33], and protein knockdown was examined using Western blot analysis [34]. Soluble proteins (30 μg) were probed with anti-Pokemon (1:500), anti-survivin, anti-p14ARF and anti-Bcl-2 antibodies (1:500) (Abcam). Loading variations were normalized against β-actin, which was identified by anti-β-actin monoclonal antibody (Sigma, St. Louis, MO, USA).

### Transient transfection and luciferase activity assay

Transient gene delivery was carried out to assess the effect of *Pokemon *on *survivin *promoter activity in MDA-MB-231 and MCF-7 cells as described previously [35,36]. Briefly, 1 × 10^5 ^cells were mixed with *survivin *promoter constructs and *Pokemon *expression vector or Pokemon siRNA#1. At 48 hours after transfection, cell extracts were prepared with 1 × lysis buffer, and a 10-μl aliquot of the supernatant was mixed with 50 μl of luciferase assay reagent (Promega) and analyzed with a Microplate Luminometer (Beckman Coulter, Fullerton, CA, USA). Luciferase activity was normalized by using a Renilla luciferase internal control.

### Chromatin immunoprecipitation assay

MCF-7 and MDA-MB-231 cells were fixed by the addition of 1% formaldehyde to the medium for 10 minutes. Formaldehyde was quenched by the addition of 1 × glycine for 5 minutes at room temperature. A chromatin immunoprecipitation (ChIP) assay was performed as described previously [37] with 3 μl of immunoprecipitated DNA and primers (forward 5'-GTC AAG CTT GCC GCC GCC GCC ACC TC-3' and reverse 5'-GTC AGA TCT TTG GGA TTA CAG GCA TGC ACC AC-3') located at -441 to -418 bp and -17 to +1 bp of the *survivin *promoter.

### Statistical analysis

Spearman's rank-correlation coefficients were used to assess the relationship between *Pokemon *and *survivin *expression, the Wilcoxon rank-sum test or the Kruskal-Wallis test were utilized with categorical variables and Kaplan-Meier survival curves were produced to examine the relationship between *Pokemon *expression and mortality. Results were considered statistically significant at *P *< 0.05.

## Results

### Overexpression of *Pokemon *in breast cancer is positively correlated with disease progression

**Figure 1:**
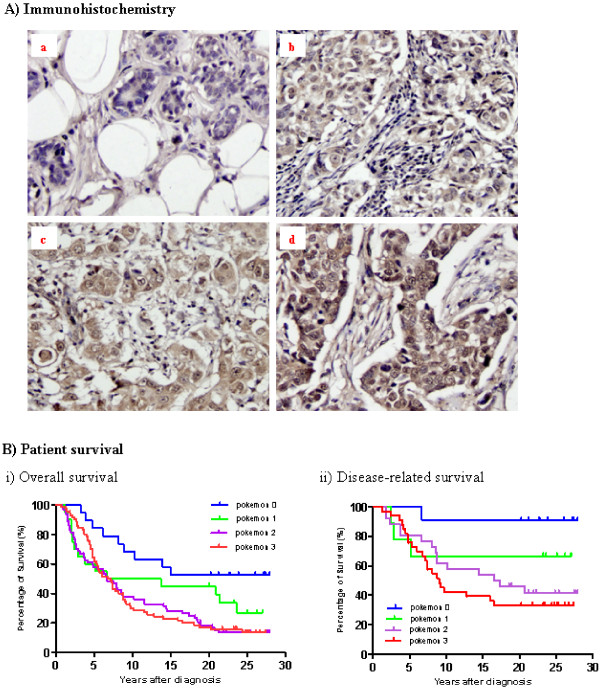
***Pokemon *expression in breast cancer tissues and its relationship with patient survival**. **(A) **Immunohistochemistry indicating *Pokemon *expression in normal and malignant breast tissues. Results were scored by a researcher blinded to the samples and by a pathologist. The images show *Pokemon *expression in **(a) **normal breast lobules and in breast cancers scored as **(b) **1, **(c) **2 and **(d) **3, respectively. **(B) **Kaplan-Meier plots of **(i) **overall survival (*n *= 175, *P *= 0.01) and **(ii) **disease-related survival (*n *= 79, *P *= 0.0134). Data are from the Yale Tissue Microarray (YTMA)-23 microarray containing 175 breast cancer cases with clinical data and more than 30 years of follow-up. In disease-related survival plots, disease-free survival or patients who died as a result of breast cancer are included.

The YTMA-23 tissue microarray, provided by the Yale Cancer Center Critical Technology Group, was evaluated for *Pokemon *expression. This array consists of 246 breast cancer cases and normal controls, among which 182 malignant tissues were histologically interpretable. The results showed that *Pokemon *was undetectable in normal breast lobules, but overexpressed in 158 (86.8%) of 182 cancerous tissues. The level of *Pokemon *expression was assessed according to the intensity of immunostaining and assigned the score of 0 to 3 (negative to most intensive) (Figure 1A). This YTMA-23 microarray came with complete clinical records and follow-up, and therefore the correlation between *Pokemon *expression and clinical pathological parameters was analyzed. As summarized in Table 1, *Pokemon *expression correlated positively with tumor size (*P *= 0.0148) and lymph node metastasis (*P *= 0.0014), but not with patient age, tumor type and nuclear grade. *Pokemon *was also found to be overexpressed in 28 (80%) of 35 interpretable recurrent breast tumors in another YTMA-89 microarray.

**Table 1:** Correlation of *Pokemon *expression with clinicopathological parameters^a^

	*Pokemon *(*n *= 182)				
		
Variables	3	2	1	0	*P *value
Subtotal, *n *(%)	72 (39.6)	69 (37.9)	17 (9.3)	24 (13.2)	
Age, yr (%)					0.2451
>50	58 (40.8)	55 (38.7)	13 (9.2)	16 (11.3)	
≤50	13 (33.3)	14 (35.9)	4 (10.3)	8 (20.5)	
Tumor types, *n *(%)					0.8733
Colloid	3 (37.5)	3 (37.5)	1 (12.5)	1 (12.5)	
Ductal	35 (47.9)	26 (35.6)	5 (6.8)	7 (9.6)	
Lobular	14 (53.8)	8 (30.8)	3 (11.5)	1 (3.8)	
Tumor size, cm^3 ^(%)					0.0148
>2	47 (42.7)	46 (41.8)	9 (8.2)	8 (7.3)	
≤2	23 (33.8)	22 (32.3)	8 (11.8)	15 (22.1)	
Node metastasis, *n *(%)					0.0014
Positive	49 (44.1)	38 (34.2)	8 (7.2)	16 (14.4)	
Negative	22 (31.9)	30 (43.5)	9 (13.0)	8 (11.6)	
Nuclear grade, *n *(%)					0.057
1	5 (23.8)	9 (42.6)	2 (9.5)	5 (23.8)	
2	40 (40.4)	38 (38.4)	12 (12.1)	9 (9.1)	
3	25 (45.5)	20 (38.4)	3 (12.1)	7 (9.1)	
ER, *n *(%)					0.429
Positive	38 (37.6)	39 (38.6)	11 (10.9)	13 (12.9)	
Negative	34 (42.0)	30 (37.0)	6 (7.4)	11 (13.6)	
PR, *n *(%)					0.3444
Positive	41 (40.2)	34 (33.3)	14 (13.7)	13 (12.8)	
Negative	31 (38.8)	35 (43.8)	3 (3.7)	11 (14.7)	
*HER2*, *n *(%)					0.2309
Positive	31 (38.7)	28 (35.0)	7 (8.8)	14 (17.5)	
Negative	41 (38.3)	41 (38.3)	11 (10.3)	14 (13.1)	

^a^Data are from the Yale Tissue Microarray-23, in which 182 breast cancer cases were histologically interpretable and analyzed for the correlation of *Pokemon *expression with clinicopathological parameters. ER, estrogen receptor; PR, progesterone receptor; HER2, human epidermal growth factor receptor 2.

Tumor size and lymph node metastasis are well-known prognostic factors for breast cancer, and therefore the effect of *Pokemon *expression on patient survival was further analyzed using Kaplan-Meier plots. As shown in Figure 1B, *Pokemon *expression was negatively correlated with overall survival (*n *= 175, *P *= 0.01) and in particular disease-related survival (*n *= 79, *P *= 0.0134) of breast cancer patients, indicating that *Pokemon *is a negative prognostic indicator.

The estrogen receptor (ER), progesterone receptor (PR) and human epidermal growth factor receptor 2 (*HER2*) genes are well-established biomarkers and therapeutic targets for breast cancer. The correlation of *Pokemon *expression with these three molecular markers was assessed using Kruskal-Wallis tests, and the results showed that *Pokemon *expression did not correlate with ER, PR or *HER2 *alone or with any combination of the three genes.

### Survivin is a downstream target of *Pokemon*

**Figure 2:**
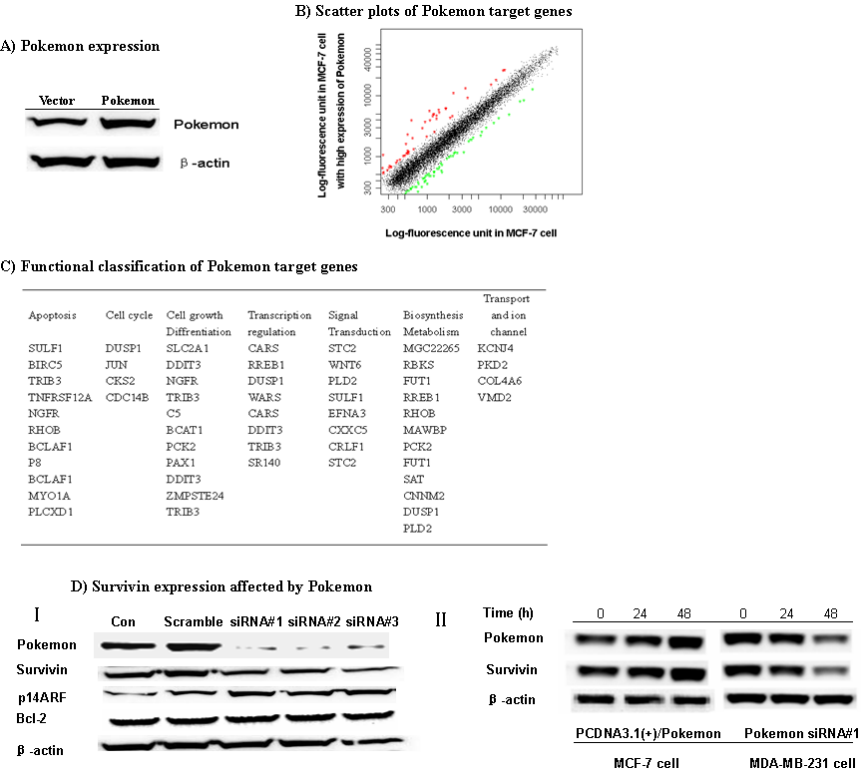
**Target genes of Pokemon in MCF-7 cells**. cDNA microarray analysis was performed as described in Materials and methods. Data from three independent replicates were combined, and any spot with at least a 2.0-fold increase or decrease compared to vector control was considered a significant change. Signaling pathways were classified by using Molecule Annotation System (MAS) 2.0 software (CapitalBio, Beijing,China ). **(A) **Ectopic expression of *Pokemon *in MCF-7 breast cancer cells detected by Western blot analysis. **(B) **Scatterplots showing differential expression profile of *Pokemon *target genes in MCF-7 cells. **(C) **Pathway classification of *Pokemon *target genes in MCF-7 cells. **(D) ***Survivin *expression regulated by Pokemon. MDA-MB-231 cells treated with siRNA for 48 hours were harvested for Western blot analysis with the indicated antibodies **(I)**. Cells transiently transfected with either *Pokemon *expression vector (MCF-7 cells) or siRNA (MDA-MB-231 cells) were used for time-dependent expression by Western blot analysis as described in Materials and methods **(II)**.

The positive correlation of *Pokemon *expression with disease progression suggests that *Pokemon *may promote cancer development. To determine the underlying mechanisms, we attempted to identify genes regulated by Pokemon in MCF-7 breast cancer cells. A total of six MCF-7 cell clones with stable *Pokemon *overexpression were isolated (Additional file 2), and three of them were subjected to cDNA microarray analysis. Figure 2 shows representative data of microarray analysis, in which a total of 121 genes were found to have more than a twofold increased or decreased expression in *Pokemon*-overexpressing cells compared to the vector control. On the basis of the Kyoto Encyclopedia of Genes and Genomes and the Gene Ontology databases, these genes are classified into seven signaling and/or metabolic pathways (Figure 2C). Notably, these *Pokemon *target genes are mainly involved in apoptosis, cell cycle, differentiation and biosynthesis, suggesting the importance of *Pokemon *in cell growth, proliferation and carcinogenesis.

Interestingly, *BIRC5 *(*survivin*), a negative prognostic factor for breast cancer [38], was found to be one of the downstream targets of Pokemon. Therefore, transfection experiments were performed to determine the role of Pokemon in inducing the expression of *survivin*. MCF-7 cells were transfected with *Pokemon *expression vector to increase the level of *Pokemon*, while MDA-MD-231 cells were transfected with Pokemon siRNA to knock down its expression. As shown in Figure 2D(I), three different siRNA targeting 426 to 444 bp, 476 to 494 bp and 624 to 642 bp of *Pokemon *mRNA successfully knocked down its expression and in turn led to a decrease of *survivin*, proving the cDNA microarray data. A time-course study showed that significant silencing of *Pokemon *by siRNA was observed at 48 hours after transfection. Interestingly, *Pokemon *silencing in MDA-MD-231 led to upregulation of p14ARF, but had no effect on Bcl-2 expression. In addition, transient expression of *Pokemon *in MCF-7 cells induced *survivin *upregulation in a parallel manner to *Pokemon *levels (Figure 2D(II)), supporting the cDNA microarray data from stable clones. Taken together, these results suggest that Pokemon indeed induces *survivin *expression.

### Survivin and *Pokemon *expression is highly correlated in human breast cancer tissues

**Figure 3:**
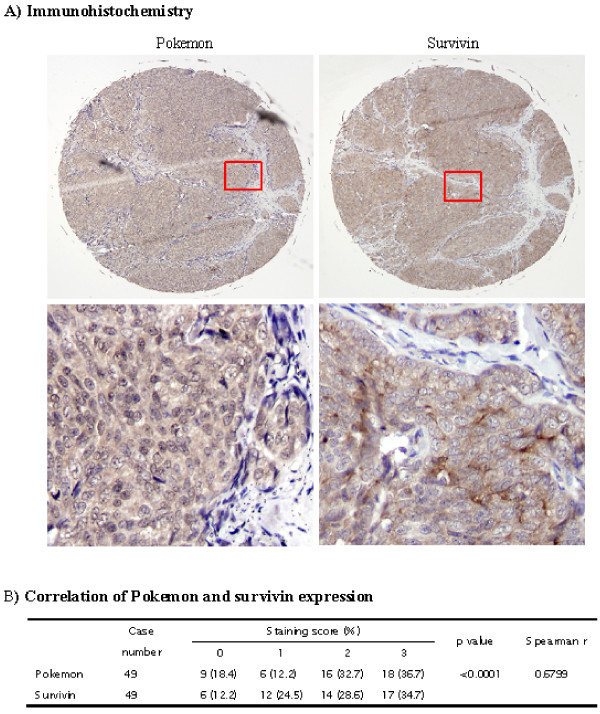
**Correlation of *survivin *and *Pokemon *expression in breast cancer tissue**. *Pokemon *and *survivin *expression in breast tissues was examined by immunohistochemistry as described in Materials and methods. **(A) **Images show *Pokemon *and *survivin *expression in two adjacent sections, which are histologically similar but not the same. Red boxed regions are amplified in lower panels. **(B) **Summary of *Pokemon *and *survivin *expression data. Spearman's rank-correlation coefficients were used to test the relationship between *Pokemon *and *survivin *expression.

To confirm that the induction of *survivin *by Pokemon indeed occurs in breast cancer, adjacent tissue microarrays containing 50 breast cancer tissue samples and matching adjacent normal breast tissue samples were subjected to immunohistological analysis of *Pokemon *and *survivin *expression. As shown in Figure 3, the levels of *survivin *and *Pokemon *expression were found to be highly correlated (*r *= 0.6799, *P *< 0.0001). In normal breast tissues, the expression levels of both *Pokemon *and *survivin *were low (data not shown).

### *Pokemon *regulates *survivin *expression via DNA sequence-specific binding to its promoter

**Figure 4:**
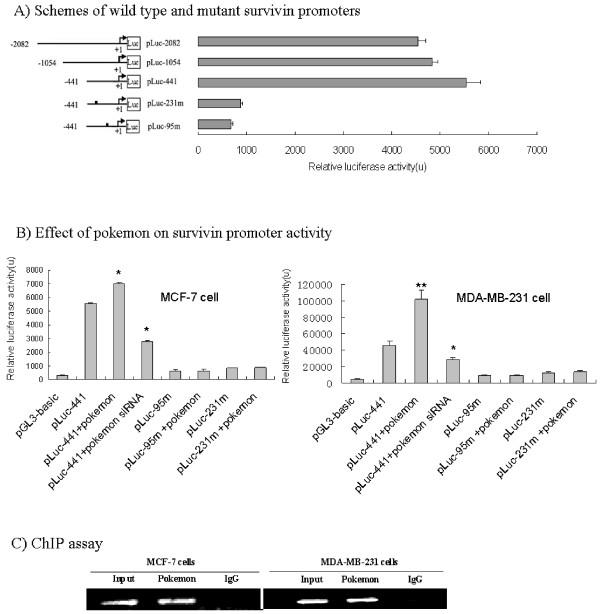
**Pokemon stimulates *survivin *expression by direct binding to its promoter**. **(A) **Schematic representation of survivin promoter-luciferase reporter plasmids, including wild-type *survivin *promoter and 5' progressive deletions, as well as site-directed mutants at Pokemon binding sites, pLuc-95 m (at -95 bp) and pLuc-231 m (at -231 bp). Refer to Materials and methods for the plasmid construction. Survivin promoter activity was analyzed in MCF-7 cells by luciferase assay. **(B) **Regulation of survivin promoter activity by Pokemon in MCF-7 and MDA-MB-231 breast cancer cells. Wild-type and mutant promoter (pLuc-441) was cotransfected with *Pokemon *gene or Pokemon small interfering RNA siRNA#1, and luciferase activity was measured with β-galactosidase as an internal control. Data represent the mean of three independent experiments ± SD. Statistical significance was tested using Student's *t*-tests with *P < 0.05 and **P < 0.01. **(C) **Chromatin immunoprecipitation assay indicating that Pokemon bound to survivin promoter in MCF-7 and MDA-MB-231 breast cancer cells. Rabbit immunoglobulin G was used as a negative control. Refer to Materials and methods for details.

We further explored the mechanism by which *Pokemon *regulates the expression of *survivin*. The promoter region of *survivin*, containing 2,080 bp upstream of the transcription start site, was cloned and used to drive the expression of luciferase reporter in MCF-7 and MDA-MB-231 breast cancer cells. Progressive deletions of the 5' end of the *survivin *promoter revealed that the -441-bp fragment possesses full promoter activity (Figure 4A). Promoter motif analyses recognized two GT boxes (GGGTG), located at -231 to -227 bp and -95 to -91 bp, which are potential binding sites of Pokemon. Therefore, this promoter fragment was used to investigate the regulatory role of Pokemon on its activity. As shown in Figure 4B, cotransfection of PCDNA3.1(+)/Pokemon greatly increased the activity of -pluc-441 in both MCF-7 and MDA-MB-231 cells, while the delivery of Pokemon siRNA significantly reduced its basal activity, suggesting Pokemon as a positive regulator of survivin promoter. Furthermore, replacing GGGTG with AAAAA in these two GT boxes of pluc-441 (luciferase plasmid at -441 bp) abrogated the promoter's responsiveness to Pokemon in both MCF-7 and MDA-MB-231 cells (Figure 4B), and interaction between Pokemon and *survivin *promoter was further confirmed by ChIP assay. As shown in Figure 4C, *survivin *promoter fragment was amplified by polymerase chain reaction assay from chromatin precipitated by an anti-Pokemon antibody, but not by nonspecific immunoglobulin G. These data suggest that Pokemon upregulates *survivin *expression through direct binding to the GT boxes in its promoter.

## Discussion

Pokemon is an oncogenic transcription factor. Embryonic fibroblasts (MEF) from *Pokemon*-null mice are resistant to cellular or viral oncogene-induced carcinogenic transformation. On the other hand, ectopic expression of *Pokemon *makes the MEF cells susceptible to oncogenic transformation. These findings suggest an important role of *Pokemon *in carcinogenesis [10]. In this study, we have shown that *Pokemon *was overexpressed in primary and recurrent breast cancer tissues and upregulated the expression of anti-apoptotic survivin, leading to disease progression and poor survival.

Using tissue microarray technology, we have shown that *Pokemon *was overexpressed in 86.8% of breast cancer tissue, but not in normal beast tissue, indicating its tumor-specific expression. More importantly, *Pokemon *expression correlated positively with tumor size and lymph node metastasis, both of which are indicators of unfavorable clinical outcome. Indeed, the level of *Pokemon *expression was negatively correlated with survival rate. These findings indicate that *Pokemon *may be a novel prognostic marker for breast cancer and a potential therapeutic target. We therefore attempted to elucidate the underlying mechanism of its tumor-promoting function.

Previous studies have shown that Pokemon functions as an oncoprotein by inhibiting the ARF/p53 pathway [10]. Using cDNA microarray analyses, we have shown that in breast cancer cells, Pokemon regulates the expression of at least 121 genes, some of which are involved in important cellular signaling/metabolic pathways. *Survivin*, an antiapoptotic protein and a negative prognostic indicator of breast cancer [38,39], is one of those genes whose expression was induced by Pokemon. Our study on breast cancer tissues from 50 patients (Figure 3) showed that the expression levels of *survivin *and *Pokemon *were highly correlated (*P *< 0.0001, *r *= 0.6799), suggesting that Pokemon serves as an upstream inducer of *survivin *in breast cancers.

**Figure 5:**
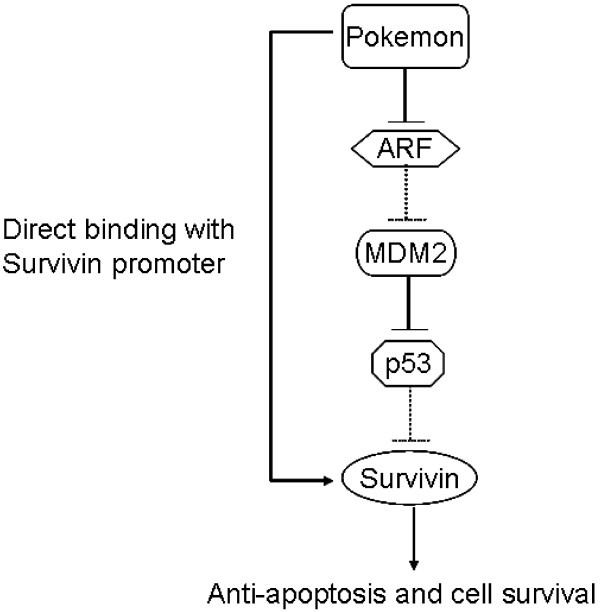
**Hypothetic pathways by which Pokemon regulates *survivin *expression**. p53 suppresses *survivin *expression and induces cell apoptosis or senescence [28,29]. Pokemon activates survivin signaling by suppressing the ARF/p53 pathway [1]. The present study shows that Pokemon also enhances *survivin *expression via direct binding to its promoter, promoting breast oncogenesis.

Survivin is widely implicated in cell carcinogenesis, tumor progression and resistance to radiation therapy and chemotherapy [30,40]. Previous studies showed that p53 is a suppressor of *survivin *expression and that loss of p53 function would lead to the induction of *survivin*, resulting in cancer growth and resistance to chemotherapeutic agents [28,41]. Pokemon has been shown to repress the expression of p53 [10,42]. Therefore, Pokemon may induce the expression of *survivin *indirectly by suppressing p53. However, in this study, we have demonstrated that Pokemon can directly induce the expression of *survivin *by binding to the GC boxes in its promoter. This finding reveals a new signaling pathway of Pokemon-mediated oncogenesis and advances understanding of *survivin *regulatory mechanisms (Figure 5).

In summary, we have found that *Pokemon *is overexpressed specifically in breast cancer tissue, but not in normal breast tissue. Its oncogenic function may be partly due to its ability to directly induce the expression of *survivin*, an important cancer-promoting gene. The correlation of *Pokemon *expression with tumor size, lymph node metastasis and poor patient survival suggests its potential role as a prognostic marker and therapeutic target for the treatment of this disease.

## Conclusions

*Pokemon *was overexpressed in primary and recurrent breast cancer tissue and was correlated with tumor size, lymph node metastasis and poor patient survival, making it a potential target for the treatment of this malignancy. *Pokemon *might prompt breast cancer progression through upregulating the expression of *survivin*, an important cancer-promoting gene.

## Abbreviations

ChIP: chromatin immunoprecipitation assay; EGFR: epidermal growth factor receptor; FBI: factor that binds to inducer of shot transcripts 1; HER2: human epidermal growth factor receptor 2; IAP: inhibitor of apoptosis protein; KLF5: Krüppel-like factor 5; LRF: leukemia/lymphoma-related factor; PMSF: phenylmethanesulfonyl fluoride.

## Competing interests

The authors declare that they have no competing interests.

## Authors' contributions

ZX and JYY designed the study and drafted the manuscript. ZX, MJ, LH, LF, TC, YL, WJ and XZ performed the study. CD procured the tissue microarrays and revised the paper. All authors read and approved the final manuscript.

## Supplementary Material

Additional file 1 **Descriptive statistics of tissue microarray**. Yale Tissue Microarray (YTMA)-23 contained 246 breast cancer cases with clinical records. The descriptive statistical data are summarized below. Click here for file

Additional file 2 **MCF-7 cell clones with stable overexpression of *Pokemon***. MCF-7 cells were transfected with pcDNA3.1/Pokemon or the empty vector, and the transfected cells maintained with neomycin were subjected to Western blot analysis for the detection of *Pokemon *expression. Click here for file
