# Supplementary material for: Pro-oncogene Pokemon promotes breast cancer progression by upregulating survivin expression
Source: Breast Cancer Res. 2011 Mar 10;13(2):R26. doi: 10.1186/bcr2843 (PMC3219187; doi:10.1186/bcr2843)
Supplement: Additional file 1 — Descriptive statistics of tissue microarray. Yale Tissue Microarray (YTMA)-23 contained 246 breast cancer cases with clinical records. The descriptive statistical data are summarized below. [file bcr2843-S1.DOC]

**Supplementary Table 1. Descriptive statistics**

**Tissue microarray, YTMA-23, contained 246 breast cancer cases with clinical records. Summarized below are the descriptive statistical data.**

| **Variables** | **Descriptive Statistical Data (%)** |
| --- | --- |
| **Age at diagnosis** | |
| Mean | 59.7 |
| **Node status** |  |
| Positive | 120 (54.5) |
| Negative | 100 (45.5) |
| **ER expression** |  |
| Positive | 114 (51.8) |
| Negative | 106 (48.2) |
| **PR expression** |  |
| Positive | 116 (52.7) |
| Negative | 104 (47.3) |
| **HER-2 expression** |  |
| Positive | 84 (38.2) |
| Negative | 136 (61.8) |
| **Pathology** |  |
| Infiltrating duct carcinoma | 201 (91.4) |
| Infilt.ductular carcinoma | 11 (5) |
| Carcinoma,NOS | 8 (3.6) |
| Medull.ca.with lymph.sroma | 5 (2.3) |
| Mucinous adenocarcinoma | 5 (2.3) |
| **Tumor grade** |  |
| 1 | 28 (12.7) |
| 2 | 115 (52.3) |
| 3 | 71 (32.3) |
| N/A | 6 (2.7) |
| **Tumor size** |  |
| >4 cm | 43 (19.5) |
| 3-4cm | 36 (16.4) |
| 2-3cm | 54 (24.5) |
| 1-2cm | 54 (24.5) |
| <1cm | 29 (13.2) |
| N/A | 4 (1.9) |
