# Supplementary figures and images for: Pro-oncogene Pokemon promotes breast cancer progression by upregulating survivin expression
Source: Breast Cancer Res. 2011 Mar 10;13(2):R26. doi: 10.1186/bcr2843 (PMC3219187; doi:10.1186/bcr2843)

**Figure S1**

**
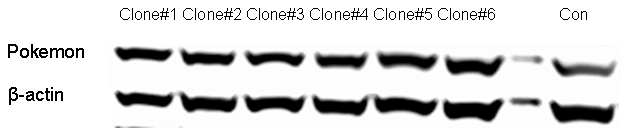
**

Supplement: Additional file 2 — MCF-7 cell clones with stable overexpression of Pokemon. MCF-7 cells were transfected with pcDNA3.1/Pokemon or the empty vector, and the transfected cells maintained with neomycin were subjected to Western blot analysis for the detection of Pokemon expression. [file bcr2843-S2.DOC]
